# Comprehensive application of high tibial osteotomy, chronic distraction tissue regeneration, and computer-assisted external fixation in the treatment of severe knee osteoarthritis: A case report

**DOI:** 10.1097/MD.0000000000018636

**Published:** 2020-01-24

**Authors:** Weiye Zhang, Chunyou Wan, Tao Zhang, Mingjie Wang, Zhao Liu, Ningning Zhang, Yuanhang Zhao

**Affiliations:** a Tianjin University of Traditional Chinese Medicine; b Tianjin Hospital, Tianjin, China.

**Keywords:** case report, external fixation, genu varus, high tibial osteotomy, knee osteoarthritis

## Abstract

**Rationale:**

Knee osteoarthritis (KOA) is a common disease. It has long been believed that the main causes of KOA are knee degenerative diseases, trauma, overwork, and labor habits. However, long-term deformity leads to uneven stress on the surface of the knee joint, and the cause of lower limb force line damage has not been taken seriously. Comprehensive application of high tibial osteotomy (HTO), chronic distraction tissue regeneration, and computer-assisted external fixation for the treatment of severe KOA has many advantages over total knee arthroplasty, such as lasting and thorough orthopedic effects, a lower cost, and a faster recovery.

**Patient concerns:**

The patient was a 48-year-old male with KOA caused by long-term genu varus, resulting in pain in both knees, especially in the right knee. The right knee pain had been aggravated for 2 years, and he was admitted to the hospital for left knee pain for 1 month.

**Diagnoses:**

X-ray: The patient has right KOA and varus deformity

**Interventions:**

Comprehensive application of HTO, chronic distraction tissue regeneration technology, and computer-assisted external fixation technology has a good therapeutic effect for patients with KOA and varus.

**Outcomes:**

The patient's severe genu varus was corrected, the bone and soft tissue regeneration was good, the lower limb force line was improved, lower limb function was restored well, and the treatment was satisfactory.

**Conclusion:**

For the treatment of KOA patients with genu varus, the combination of HTO, chronic distraction tissue regeneration, and computer-assisted adjustment of external fixation technology have a good effect on the correction of genu varus deformity and the recovery of the lower limb force line. This treatment method is also conducive to preventing postoperative infection and avoiding secondary trauma caused by the removal of internal fixation plates.

## 1 Introduction

Knee osteoarthritis (KOA) is a common clinical disease that most commonly affects adults aged 65 and older. Lawrence et al^[1]^ reported a prevalence rate of 33.6% in the United States as well as a higher prevalence in women than in men. Currently, the field of medicine has come to realize that KOA is caused by the complex interaction between structural and mechanical factors, including joint integrity, genetic susceptibility, local inflammation, mechanical force, and cell and biochemical factors.^[2]^ Therefore, it has long been believed that the main causes of KOA are knee degenerative diseases, trauma, overwork, postural errors, and so on. However, knee deformities, representing one of the important causes of KOA, have not been taken seriously. Knee deformities can cause knee cartilage surface wear, lower limb force line damage, and tibial plateau collapse, ultimately leading to KOA. At present, the most important treatment method in the clinic is knee arthroplasty. This method is costly, traumatic, and prone to complications and artificial joint revision after surgery. However, the comprehensive application of high tibial osteotomy (HTO), chronic distraction tissue regeneration, and computer-assisted external fixation for the treatment of severe KOA has accurate and effective control of the HTO angle, low levels of surgical trauma, low bleeding volumes, postoperative adjustment ability, fast patient recovery, and many other advantages. This article introduces the case of a patient with KOA who suffered from lower limb force line damage due to a long-term varus deformity. After the comprehensive application of HTO, chronic distraction tissue regeneration technology, computer-assisted external fixation, and other methods, a good therapeutic effect was achieved. This article establishes a novel idea for the treatment of KOA.

## 2 Case introduction

### 2.1 Basic information

The patient, who was a 48-year-old male with a height of 17 cm and a weight of 93.6 kg, had suffered from intermittent pain of the right knee joint for 2 years and was admitted to the hospital for 1 month with aggravated pain of the left knee joint. X-ray: Osteoarthritis and varus deformity of both knees. Magnetic resonance imaging showed that the patient had grade IV cartilage softening in the medial femoral and tibial joints of the right knee, cartilage softening in the right patellofemoral joint, a prominent medial meniscus of the right knee, irregular abrasions and grade III degeneration in the body, degeneration and grade III injury in the anterior and posterior horns of the right knee, synovitis of the right knee joint, effusion of the articular capsule, and formation of a free body in the anterior intercondylar region. In addition, the following parameters were found: right knee ROM >120 degrees, WOMAC score = 80 points, and Caton–Deschamps index = 0.8. Low-grade medial compartment OA (<Ahlback II) without involvement of the lateral compartment or patellofemoral compartment was found. Using the method proposed by Moreland et al to measure the right tibiofemoral angle of the patient, the angle was found to be 179 degrees.

### 2.2 The preoperative planning method

When patients are young, when they suffer from KOA, when medial single compartment degeneration is serious, and when there is knee varus, doctors recommend retaining the knee joint via HTO. At the time of presentation, the main methods for the clinical treatment of this patient could be divided into 2 types: knee arthroplasty and HTO. Considering that the patient was 48 years old and young at the time, it was considered that this patient with knee arthroplasty may also have a low quality of life due to knee pain and may experience readmission after a few years. Because the basic condition of the patient was more in line with the indications for HTO, we believed that HTO was the preferred surgical method and that total knee arthroplasty should be used as an alternative method. Before the operation, the patient received a detailed explanation, underwent self-selection and ultimately chose HTO surgery.

### 2.3 Surgical procedure

First operation: after the anesthesia came into effect, the patient was subjected to a proximal anteromedial arc incision of the right leg. The incision was made from the anteromedial part of the upper tibia to the lower edge of the plateau, approximately 8 cm in length. The skin, subcutaneous tissue and superficial and deep fascia were cut to expose the goose foot and the posterior tibial edge. At the level of the upper margin of the goose foot, a round bone needle was inserted approximately 2 cm ahead of the posterior tibial margin to the proximal end of the contralateral fibular capitulum. When the perspective of the C-arm X-ray machine was satisfactory, a round bone needle was inserted into the contralateral cortex 2 cm in front of the needle. Along the osteotomy plane that was determined by 2 round pins, the tibia was cut with a bone chisel, and only a small amount of cortex was preserved on the lateral side. At an angle of 110 degrees, the tibial tubercle was osteotomized below the tubercle to connect the tubercle with the distal segment.

**Figure 1:**
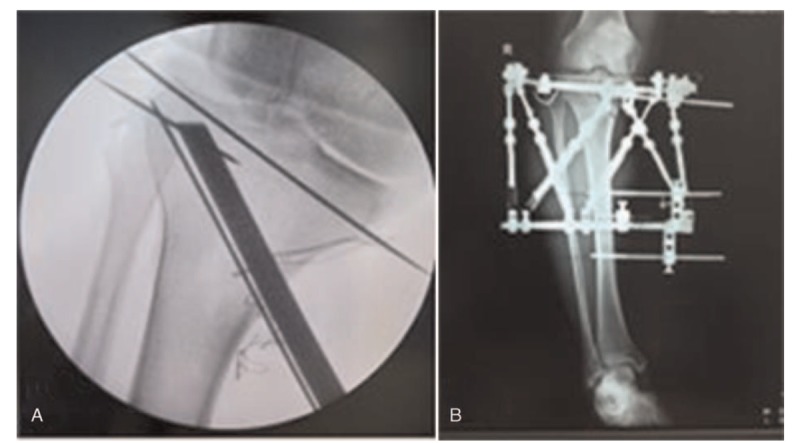
(A) X-ray of osteotomy position in HTO. (B) X-ray after installing external fixator in HTO operation. HTO = high tibial osteotomy.

With the help of the C-arm X-ray machine, the proximal tibia and the middle and lower tibia were needled, and a Taylor stent was installed. The Taylor stent was adjusted to break the residual cortex of the lateral tibia, and then the screw was fastened. X-ray examination performed during the operation showed that the tibial osteotomy angle was good, and the length of the Taylor stent position length was appropriate. The flexion and extension of the knee joint were good and reliable. The wound was closed layer by layer and dressed with a sterile dressing. The operation was successful, and the anesthesia was satisfactory. The patient bled approximately 200 mL during the operation without complaints of obvious discomfort. After the operation, he was conscious and had a good blood supply to the affected limbs. He returned to the ward safely. Figure 1: X-ray after the HTO operation and external fixator installation.

Six days after the operation, the stent was adjusted, the patient was instructed to pay attention to the orthopedic stretching exercises and involvement in the stent adjustment program; he received a regular outpatient review and was discharged.

Second admission process: physical examination: strong external fixation of the right leg stent, no exudation of external fixation pinholes, no tenderness or tapping pain in the right leg, normal flexion, and extension of the right knee joint, 20 degrees dorsal extension of the right class joint, 30 degrees plantar flexion, palpable pulsation of the dorsal pedal artery, good sensation of the toe, and good movement and peripheral blood circulation. X-ray: after external fixation of the right leg, the fixation was strong, and the fracture healed.

The second operation: after admission, the patient showed active improvement in the relevant examinations, underwent external fixation for removal of the Taylor stent, and received symptomatic treatment such as ice compression and pain relief after the operation. The wound was cleaned and dressed regularly, and the patient was discharged after improvement.

### 2.4 Postoperative recovery

**Figure 2:**
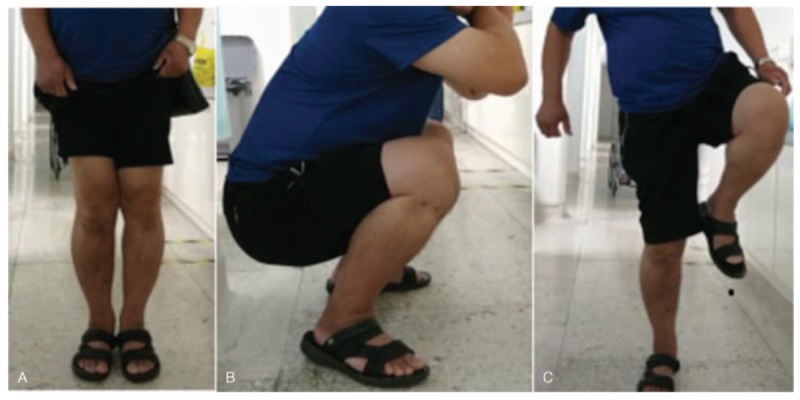
Functional recovery of limbs and joints at 12 mo after operation.

The patient remained in an external fixator for 135 days after the operation. Fracture healing was confirmed, fixation was strong, the external fixator was removed selectively, the patient was instructed to exercise moderately, and the patient was reexamined regularly. The patient was not suitable for follow-up. The patient recovered well after the operation. The recovery of knee joint function was satisfactory. After removal of the external fixator, we followed the patient for up to 6 months. The right tibiofemoral angle was 179 degrees before the operation, and the ROM of the right knee joint was 120 degrees. The right tibiofemoral angle was 170 degrees after operation, and the ROM of the knee joint was −5 degrees to 120 degrees. The HSS knee score was 100 (pain, 30; function, 22; range of motion, 18; muscle strength, 10; flexion deformity, 10; and stability, 10). After 12 months of follow-up, the patient had recovered satisfactorily. Figure 2: Functional recovery of the limbs and joints 12 months after the operation.

## 3 Discussion

For a long time, many studies have reported that HTO is effective for the treatment of varus deformity.^[3–5]^ Clinical studies have shown that HTO can change the force line of the lower limbs by adjusting the mechanical axis of the lower limbs. For young patients with degenerative changes and accompanying cruciate ligament injury, closing wedge or medial opening wedge is the preferred method for replacing knee arthroplasty.^[5]^ Many studies suggest that age 65 should be the upper age limit for HTO.^[6]^

According to the literature, ideal open wedge high tibial osteotomy patients are young (under 56 years old), have a normal weight or are slightly overweight (body mass index 25–27.5), have a range of motion of at least 120 degrees, have a flexion contracture reduced to 5 degrees, have low medial chamber OA (<Ahlback II) not involving the lateral or patellofemoral chamber, and have a Caton–Deschamps index >0.6 and a WOMAC score >45 points.^[7]^ The patient's condition basically met these requirements for HTO. The surgical method performed was HTO combined with chronic distraction regeneration. According to the physiological basis of KOA, this case reported the correction of genu varus in a patient, and a new idea for the treatment of KOA was put forward.

In terms of the choice of fixation technology after the operation, we chose the 6-axis orthodontic external fixator, namely, the Taylor space stent (TSF). The Taylor 3-dimensional space stent is an improvement from the Ilizarov stent. The plane of the different rings can be adjusted on 6 axes and can be adjusted arbitrarily in 3-dimensional space, which makes it possible to simultaneously correct complex deformities such as angulation, rotation, lateral displacement, and shortening.^[8]^ The TSF can input 6 deformity parameters, 3 frame parameters, and 4 installation parameters or assembly parameters into the computer program. The computer automatically generates electronic prescriptions. The length of the screw can be adjusting in patients according to the prescription, and the deformity can be gradually corrected. This stent meets the needs for the treatment of chronic distraction regeneration while achieving precise and quantitative slow adjustment. Compared with traditional plate internal fixation, the TSF has many advantages, as follows: the osteotomy angle can be adjusted accurately according to the imaging results after the operation, which greatly avoids an inadequate osteotomy angle and an unsatisfactory force line of the lower limbs after the operation; the theory of chronic distraction osteogenesis (DO) can minimize bone grafting or non-bone grafting; and the patient's skin condition is not ideal and will not hinder the operation process. As long as patients meet these requirements for HTO, the TSF can be chosen as the operation mode for HTO fixation.

Ilizarov put forward the biological theory of the “tension-stress rule” in the 1950s, that is, “biological tissue can stimulate cell division and tissue formation under sustained, stable, and slow traction, thus repairing various limb defects.” Ilizarov put forward the biological theory of DO on this basis, that is, under the condition of external fixation technology, the process of bone modeling and bone remodeling will produce bone hyperplasia with the change in limb distraction.^[9]^ This procedure is the main method for the treatment of congenital bone deformities, long bone defects, posttraumatic bone infections, nonunion, malunion, and so on. Under the stable mechanical force provided by the external fixator, the technique exerts the intrinsic osteogenic activity of the autologous long bone by slowly stretching the fixed rhythm (0.75–1.00 mm/d) and gradually completing new bone reconstruction.^[10,11]^ In this case, the bone defect formed after the operation was slowly regenerated with good external fixation and distraction, and a good healing effect was achieved.

**Figure 3:**
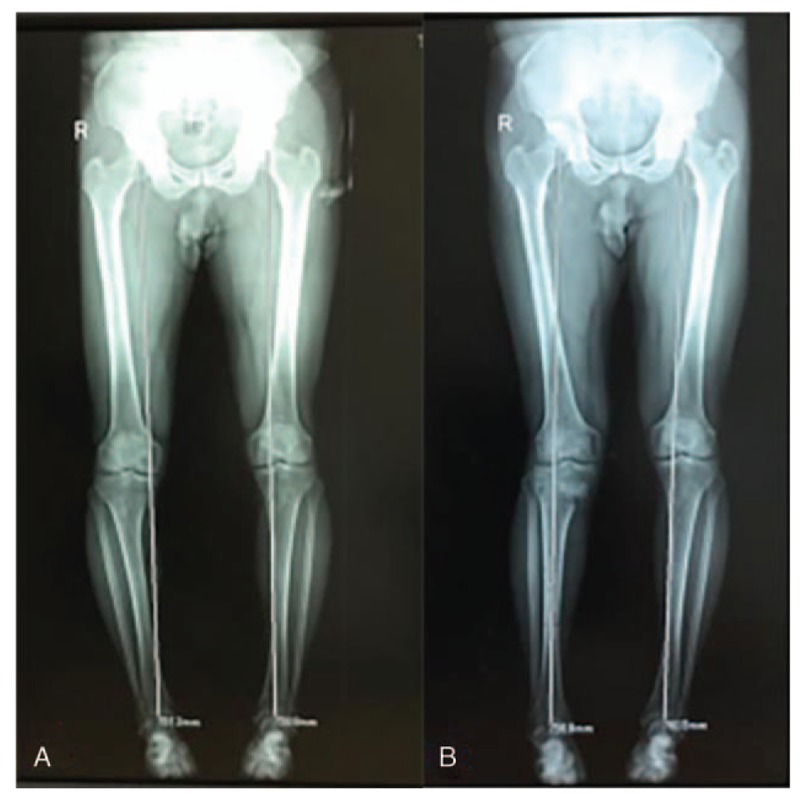
(A) Full-length image of lower limbs before operation. (B) Full-length image of lower limbs after removal of external fixator.

The ideal force line is that the center of the femoral head, the Fujisawa point of the knee joint and the center of the ankle joint form 3 points and 1 line.^[12,13]^ The patient was hospitalized 2 times within 143 days for the treatment of knee varus secondary to KOA, and ultimately, the lower limb force line was adjusted ideally. Before and after the HTO operation, we applied the full-length image of the lower limbs with the lower limb force line. This kind of image can clearly show the state of the lower limb force line, which is of great significance to the formulation of the preoperative operation plan, the effect of the operation after the operation and the judgment of the prognosis of patients. Figure 3: Comparisons of the Lower limb force line before and after the operation. After surgical correction, the right lower limb force lines form a 3-point line at the center of the femoral head, the Fujisawa point of the knee joint and the center of the ankle joint, and the genu varus deformity can be corrected. Lower extremity alignment has a major impact on the proportion of shared load between compartments of the tibiofemoral joint, and varus alignment results in a significant increase in the medial load.^[14]^ Only on the basis of correcting genu varus can patients’ KOA gradually improve.

At the same time, HTO saves the knee joint and avoids knee replacement. McNamara reports that although it is necessary to continue investigating the patient's choice and results, current studies show that HTO provides at least part of the solution for varus arthropathy patients to prolong the life of their original knee joint.^[15]^ Adjustment of Taylor external fixator with the help of a computer can correct skeletal healing deviation in time, check the degree of skeletal healing, and serve as one of the bases for the removal of the external fixator, thus providing a reference for doctors.

## 4 Conclusions

In the treatment of KOA patients with genu varus, the combination of HTO, chronic distraction tissue regeneration, and computer-assisted adjustment of external fixation technology have a good effect on the correction of genu varus deformity and the recovery of the lower limb force line. It is also conducive to preventing postoperative infection and avoiding secondary trauma caused by the removal of internal fixation plates.

## Author contributions

**Writing – original draft:** Weiye Zhang.

**Writing – review & editing:** Chunyou Wan, Tao Zhang.
